# Single-Isocenter Volumetric Modulated Arc Therapy (VMAT) Radiosurgery for Multiple Brain Metastases: Potential Loss of Target(s) Coverage Due to Isocenter Misalignment

**DOI:** 10.7759/cureus.11267

**Published:** 2020-10-30

**Authors:** Allison N Palmiero, Lana Critchfield, William St Clair, Marcus Randall, Damodar Pokhrel

**Affiliations:** 1 Radiation Medicine, University of Kentucky, Lexington, USA

**Keywords:** hyperarc, single-isocenter vmat, set up errors, multiple brain metastases, coverage loss

## Abstract

Purpose

A single-isocenter volumetric modulated arc therapy (VMAT) treatment to multiple brain metastatic patients is an efficient stereotactic radiosurgery (SRS) option. However, the current clinical practice of single-isocenter SRS does not account for patient setup uncertainty, which degrades treatment delivery accuracy. This study quantifies the loss of target coverage and potential collateral dose to normal tissue due to clinically observable isocenter misalignment.

Methods and materials

Nine patients with 61 total tumors (2-16 tumors/patient) who underwent Gamma Knife® SRS were replanned in Eclipse™ using 10 megavoltages (MV) flattening-filter-free (FFF) bream (2400 MU/min), using a single-isocenter VMAT plan, similar to HyperArc™ VMAT plan. Isocenter was placed in the geometric center of the tumors. The prescription was 20 Gy to each tumor. Average gross tumor volume (GTV) and planning target volume (PTV) were 1.1 cc (0.02-11.5 cc) and 1.9 cc (0.11-18.8 cc), respectively, derived from MRI images. The average isocenter to tumor distance was 5.5 cm (1.6-10.1 cm). Six-degrees of freedom (6DoF) random and systematic residual set up errors within [±2 mm, ±2^o^] were generated using an in-house script in Eclipse based on our pre-treatment daily cone-beam CT imaging shifts and recomputed for the simulated VMAT plan. Relative loss of target coverage as a function of tumor size and distance to isocenter were evaluated as well as collateral dose to organs-at risk (OAR).

Results

The average beam-on time was less than six minutes. However, loss of target coverage for clinically observable setup errors were, on average, 7.9% (up to 73.1%) for the GTV (p < 0.001) and 21.5% for the PTV (up to 93.7%; p < 0.001). The correlation was found for both random and systematic residual setup errors with tumor sizes; there was a greater loss of target coverage for small tumors. Due to isocenter misalignment, OAR doses fluctuated and potentially receive higher doses than the original plan.

Conclusion

A single-isocenter VMAT SRS treatment (similar to HyperArc™ VMAT) to multiple brain metastases was fast with < 6 min of beam-on time. However, due to small residual set up errors, single-isocenter VMAT, in its current use, is not an accurate SRS treatment modality for multiple brain metastases. Loss of target coverage was statistically significant, especially for smaller lesions, and may not be clinically acceptable if left uncorrected. Further investigation of correction strategies is underway.

## Introduction

Multiple brain metastases are common among cancer patients. Twenty to fifty percent of cancer patients develop brain metastases, with the most common primary malignancies being lung cancer, breast cancer, and melanoma [1]. Historically, this was treated with whole brain radiotherapy (WBRT), which resulted in normal tissue toxicities such as memory loss, hair loss, pituitary dysfunction, and diminished hearing, and positive treatment outcomes were not achieved (with 100% failure at one year) [2]. More recently, stereotactic radiosurgery (SRS) has gained popularity due to its high precision and accuracy in a single treatment, providing good tumor local control rates and sparing critical organs [3, 4]. For instance, a randomized trial from the MD Anderson Cancer Center on SRS of multiple brain metastases patients with or without WBRT demonstrated inferior neurocognitive results in the cohort who had received WBRT in addition to SRS [3]. Of the stereotactic treatment modalities for treating multiple metastases, Gamma Knife® (GK) radiosurgery is a gold standard [5]. However, GK patients face many challenges such as incredibly long treatment times, painful headframe placement, inability to affix the headframe to craniotomy sites, and difficulty arranging anesthesia time for claustrophobic patients. Though alternative treatment modalities such as robotic CyberKnife® and ring-mounted accelerators are available, long treatment can be problematic [6].

Linear accelerator (LINAC)-based SRS treatments provide an option for frameless SRS to brain metastatic patients. This began through multiple isocenter dynamic conformal arcs (DCA) or volumetric modulated arc therapy (VMAT)-based treatment planning, where each target was planned and treated individually. Isocenters were placed at the center of each tumor and setup and imaged individually, allowing for corrections of residual setup errors around each isocenter. Traditional LINAC-based SRS treatments can impede clinic workflow due to individual patient setup and cone-beam CT (CBCT) imaging of each isocenter and long treatment times. A new treatment planning and delivery approach is to treat multiple lesions simultaneously using a single-isocenter VMAT plan, allowing for increased clinic efficacy and tolerability [7]. Varian recently introduced a TrueBeam™ LINAC-based (or superior) single-isocenter VMAT platform known as HyperArc™ as a module in the Eclipse™ treatment planning system (TPS; Varian Medical Systems, Version 15.6) to mitigate all of these challenges mentioned above [6, 8, 9]. HyperArc (HA) VMAT can rapidly deliver treatment to multiple brain metastases and improve patient compliance and comfort.

However, there is a tradeoff when treating multiple targets simultaneously using a single-isocenter VMAT plan, similar to the HyperArc plan. First, small multiple brain metastases cannot be seen on a daily single CBCT to ensure proper target alignment. Instead, alignment must be made by rigid registration to bony anatomy. As a result, treatment delivery inaccuracies that could come with residual patient setup errors could increase. Second, lining up multiple brain tumors accurately using a single daily CBCT is almost impossible. Studies have shown localization and treatment delivery inaccuracy when treating multiple brain lesions using HyperArc VMAT [9-11]. For example, Sagawa et al. used CBCT registration information to induce a three-dimensional rotational setup uncertainty [9]. They concluded that non-negligible underdosing to the PTV was due to residual rotational setup errors. Although they successfully explain the presence and dosimetric effects of rotational setup uncertainties, their work excludes consideration of small translational effects in addition to the rotational errors. The present study is innovative because it includes setup uncertainties in all six-degrees of freedom (6DoF) to further quantify the treatment delivery inaccuracy of the HyperArc style single-isocenter VMAT plan. This work was performed in a clinically representative manner for the commissioning of single-isocenter VMAT treatments. The Eclipse TPS was used entirely, and isocenter misalignments were randomly induced errors to mimic representative patient positioning uncertainties for SRS treatments using an in-house script. Thus, this study's main purpose is to fully quantify the dosimetric effects resulting from clinically attainable residual patient setup errors in all 6-directions for the single-isocenter/multi-lesions technique. Relative loss of target coverage as a function of tumor size and distance from isocenter was investigated. Additionally, collateral dose to the adjacent organs-at-risk (OAR) was evaluated.

## Materials and methods

Patient images and contouring

After obtaining an Institutional Review Board (IRB) approval, nine patients with multiple brain metastases (all lung primary) were included in this retrospective study. These patients were previously treated with frame-based GK radiosurgery using high-resolution double-contrast magnetization-prepared rapid acquisition gradient echo magnetic resonance imaging (MPRAGE MRI) imaging (Siemens MAGNETOM®, 1.5T MRI System, Ferndale, USA). The MRI images were 512×512 pixels and 1 mm slice thickness with no gap between the slices. These Digital Imaging and Communications in Medicine (DICOM)MRI datasets were transferred from the GK planning station into Varian Eclipse TPS for contouring and planning. The target volumes were delineated by an experienced radiation oncologist on the MRI, and the gross tumor volumes (GTVs) were defined by the visible tumor in the MRI images. The planning target volumes (PTVs) were created using a uniform 1.0 mm margin around each GTV. There were 2-16 lesions per patient, with a total of 61 lesions. The tumor characteristics are summarized in Table 1. The OAR’s were delineated, including optics apparatus (both optics nerves plus chiasm), brainstem, eyes and lenses, normal brain (brain minus PTVs), and hippocampi (left and right hippocampus). The hippocampi contours were done following the Radiation Therapy Oncology Group (RTOG)-0933 atlas [12]. Since no planning CT images were available for these patients, for treatment planning purposes, a homogenous medium was assigned to the body contour in Eclipse TPS with a CT value of 0. Distance to isocenter was calculated by finding the coordinates of the PTV geometric center and determining the distance from the isocenter coordinates for each lesion.

**Table 1:** Main tumor characteristics of the patients included in this study. STD - standard deviation; GTV - gross tumor volume; PTV - planning target volume

Parameters	Mean ± STD (range)
Total tumors (n = 9 patients)	61 (2–16/patient)
GTV (cc)	1.06 ± 1.85 (0.03–11.5)
PTV (cc)	1.88 ± 2.86 (0.11–18.8)
Prescribed dose to each lesion	20 Gy in 1 fraction (70-80% isodose line)
Isocenter to tumors distance (cm)	5.50 ± 1.80 (1.58–10.15)
Tumor location (bi-lateral brain)	(all patients)
Normal brain (cc) = whole brain minus PTVs	1517 ± 198 (1213–1705)

Original VMAT plans

HyperArc (HA) style single-isocenter VMAT SRS plans were generated in the Eclipse TPS for the Truebeam LINAC (Varian Medical Systems, Palo Alto, USA) with standard millennium MLCs and 10 megavoltages (MV) flattening-filter-free (FFF) beam (maximum achievable dose rate of 2400 MU/min). The plans mimicked HyperArc VMAT geometry using 4-5 noncoplanar arc arrangements, replicating gantry rotation and couch kicks. Isocenter was placed at the approximate geometric center of all the tumors. The collimator angles were chosen manually to minimize island blocking and dose outside of the target. The dose was 20 Gy to each lesion prescribed to the 70-80% isodose line. The plans were optimized so that 95% of each PTV received at least 100% of the prescription dose with the hotspot at the center of the GTV. The dose was calculated with Anisotropic Analytic Algorithm (AAA) (Eclipse, version 15.5) with a 1.25 mm calculation grid size. Inverse optimization was performed with the photon optimizer (PO) MLC algorithm with individual dose steering ring structures to each target. Jaw tracking and normal tissue objective (NTO) was used to control the dose fall-off outside of each target for better dose conformity and to spare adjacent OAR. Planning objectives followed RTOG-0933 guidelines for hippocampal sparing that were converted to SRS dose constraints using biological effective dose, allowing maximal dose to the hippocampus to be < 6.5 Gy [12, 13]. Other OAR followed Quantitative Analyses of Normal Tissue Effects in the Clinic (QUANTEC) guidelines for single fraction treatments such as maximal dose to optic apparatus < 8.0 Gy [14]. The average beam-on time for original VMAT plans was recorded 5.20 ± 0.97 min (range, 3.01-6.02 min). Beam-on time was calculated by taking the total monitor units divided by the maximum dose rate setting of 2400 MU/min for 10 MV-FFF beam.

Simulated VMAT plans

A novel in-house method was developed to simulate clinically realistic residual setup errors by inducing uncertainties within a range of ±2 mm and ±2° in all 6DoF. These induced uncertainties were chosen randomly to mimic prospective daily CBCT setup errors obtained at the machine in a clinically realistic setting based on previous SRS patient’s data. The residual translational errors were defined for isocenter displacements. The rotational errors were defined for patient rotations relative to the isocenter around the right-left (pitch), anterior-posterior (yaw), and superior-inferior (roll) directions. After the single-isocenter VMAT plans were generated, the patient MRI images were duplicated and co-registered to the original MRI. The image registration DICOM file was exported from the Eclipse TPS and imported into an in-house MATLAB® script that generated random rotational (Δα, Δβ, Δγ) and translational (Δx, Δy, Δz) errors within [±2°, ±2 mm] in each direction. These matrices were then applied to the reference frame, and the output was a new image registration file with a simulated shift in 6DoF. The image registration DICOM file was imported back into the Eclipse TPS with the new transformation matrix applied to the registered MRI images. The single-isocenter VMAT plan was then overlaid on to the new registered image, and the dose was re-calculated with the only difference in the plans being the shift in the isocenter. For better statistics, each patient’s original plan was simulated 10 times, and all the output data averaged. A visual scripting tool (Varian Medical Systems, Palo Alto, USA) was used to export relevant dosimetric parameters for plan comparison. This process provides all dosimetric parameters, including OAR doses, using the same dose calculation algorithm as the original plan.

This same method was used to systematically induce setup uncertainties. Instead of randomly generating the translational and rotational matrices, they were set to [±0.5 mm, ±0.5°]; [±1 mm, ±1°] and [±2 mm, ±2°] in each direction, systematically. With these systematic errors, the image registration was then repeated, and the original single-isocenter VMAT plan was overlaid on to the new image set for comparison, as described above.

Plan comparison and data analysis

The simulated VMAT plans with isocenter misalignment were compared to the original single-isocenter VMAT plans. The visual scripting tool exported dose-volume histogram (DVH) parameters were used to evaluate the plan quality. The minimum, mean and maximum doses to GTV were evaluated between the plans. The PTV coverage was assessed by comparison with original PTV D95% coverage. The OAR’s were evaluated using maximal doses to hippocampi, brainstem, and optics apparatus. Dose to the normal brain was assessed using mean brain dose (MBD), V12, and V16 [15]. For each target, heterogeneity index (HI), Paddick conformation number (CN), and under-treatment ratio (UR) were evaluated [16]. The HI is the ratio between the maximum dose in the target (Dmax) and the prescription isodose (DRX). A HI value of less than 2.0 meets protocol guidelines. The Paddick CN is defined as the ratio of the target volume covered by the prescription isodose (TVRX) to the product of the target volume (TV) and the total volume covered by the prescription isodose (VRX). This parameter accounts for the position of the prescription isodose relative to the target volume. Additionally, the Paddick CN was evaluated via the under-treatment ratio (UR), where a value of 1.0 represents a fully covered or overly treated lesion, and less than 1.0 represents an undertreated lesion. Loss of target coverage for both randomly generated and systematically induced setup uncertainties was evaluated as a function of target volume and distance to isocenter. A paired two‐tail student’s t‐test (Microsoft Excel, Microsoft Corp., Redmond, USA) was used to compare the data for the original VMAT vs. simulated VMAT plans for all dosimetric parameters of target coverage and to the OAR. A value of p < 0.05 was used as a cutoff for statistically significant.

## Results

Simulated random errors

The target coverage analysis for all nine patients (61 lesions) randomly simulated 10 times each is shown in Table 2. Simulated VMAT plans showed an average loss of PTV coverage of 21.5 ± 13.6% (range, 0.4-94.7%) compared to the original VMAT plans. After applying clinically observable random transformations, a major loss in PTV coverage, CN, UR, and HI were observed. The severe loss in the average values of CN (p < 0.001) and UR (p < 0.001) for the simulated VMAT plans suggests that the prescription isodose volume did not cover the PTV as planned originally. The minimum and mean doses to GTVs were decreased by an average of 3.6 Gy and 1.5 Gy, signifying the underdosing of the GTVs.

**Table 2:** Analysis of the loss of target coverage for the original VMAT plans Mean ± STD (range) and p-values were reported for the original VMAT and simulated VMAT plans. Significant values are p < 0.05. VMAT - volumetric modulated arc therapy; STD - standard deviation; CN - Paddick conformation number; UR - under-treatment ratio; HI - Heterogeneity index; GTV - gross tumor volume; PTV - planning target volume

Target (s)	Parameter	Original VMAT plans	Simulated VMAT plans	p-value
GTVs	Max dose (Gy)	25.4 ± 0.5 (24.5–26.1)	25.3 ± 0.51 (24.3–26.1)	p = 0.385
	Min dose (Gy)	21.9 ± 0.65 (20.8–23.3)	18.3 ± 2.2 (14.3–21.3)	p < 0.001
	Mean dose (Gy)	24.0 ± 0.47 (23.2–24.8)	22.6 ± 1.4 (19.8–24.6)	p < 0.001
PTVs	% Volume covered by Rx dose (%)	98.7 ± 1.4 (95.0–100.0)	77.2 ± 13.7 (5.0 –99.7)	p < 0.001
	CN	0.70 ± 0.11 (0.35–0.91)	0.43 ± 0.18 (0.04–0.89)	p < 0.001
	UR	0.95 ± 0.15 (0.13–0.913)	0.75 ± 0.16 (0.13–1.0)	p < 0.001
	HI	1.3 ± 0.03 (1.1–1.3)	1.2 ± 0.04 (1.0–1.3)	p = 0.04

**Figure 1:**
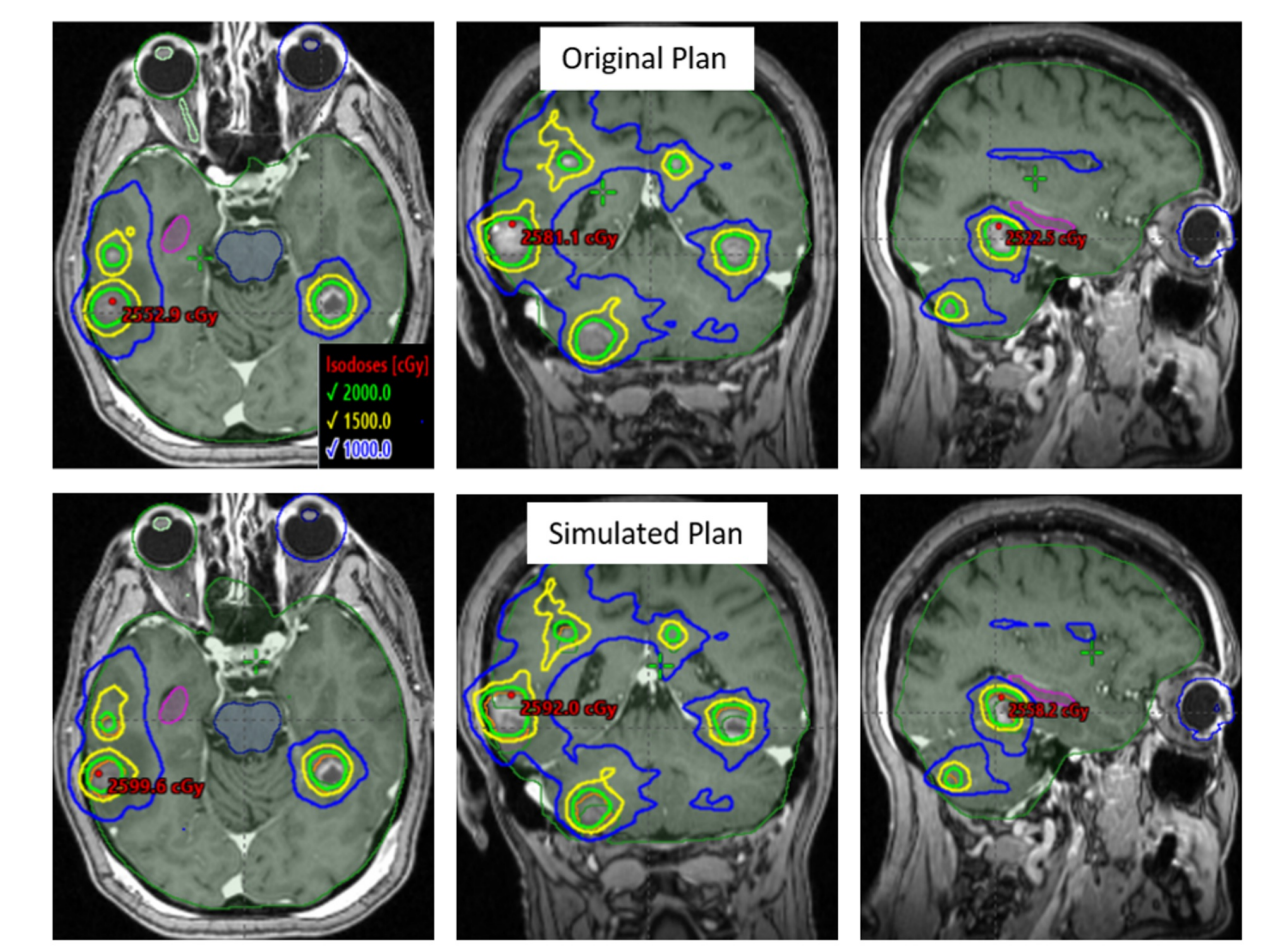
An axial, coronal and sagittal view of an example case with 16 tumors Original VMAT plan (upper panel) and simulated VMAT plan (lower panel) with randomly induced residual setup errors of ±2.0 mm and ±2.0 deg in each direction. The prescription isodose line (green) conformed to each lesion as in the original plan. Loss of target coverage is seen in the simulated VMAT plan (see lower panel) and shifted the higher isodose line closer to the hippocampus. VMAT - volumetric modulated arc therapy

Figure 1 shows an axial, coronal, and sagittal view of the original and simulated VMAT plans for an example patient with 16 tumors (not all tumors are seen). With induced random setup uncertainties within [-2.0, +2.0] mm and [-2.0, -2.0] degrees in all 6DoF, major loss of both GTV and PTV coverage was observed. In the simulated VMAT plan, loss of target coverage was evident due to the visible difference in the overlap of the PTV (orange) and the prescription isodose lines (green).

**Figure 2:**
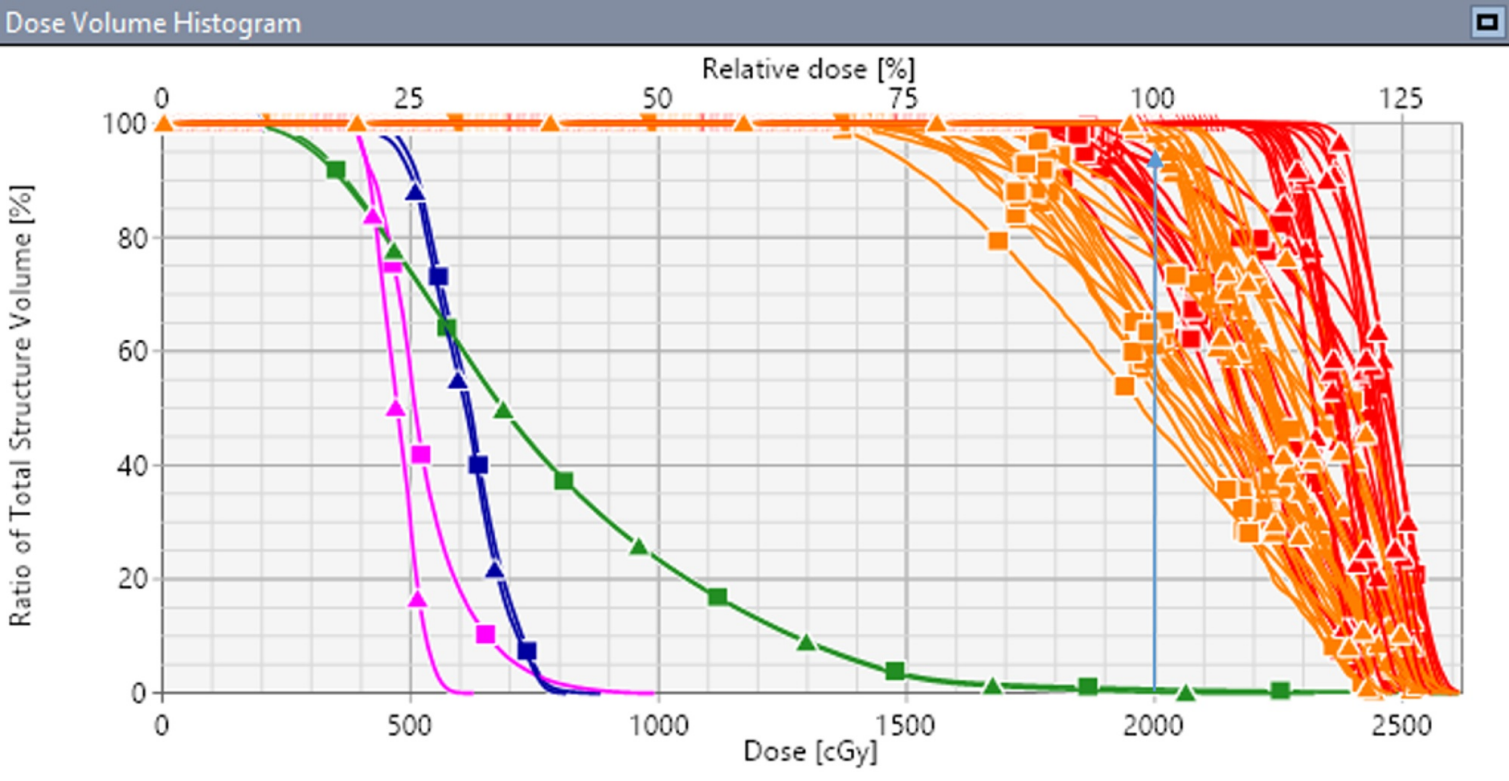
Comparison of dose-volume histogram (DVH) for the original VMAT plan (triangle) and the simulated VMAT plan (square) with randomly induced residual set up error in 6DoF The vertical blue arrow shows the original planned coverage to all PTV. Brainstem (blue) and normal brain (green) are shown. Due to small, clinically observable residual patient setup errors, unacceptable loss of target coverage was observed along with increased dose to hippocampi (pink). VMAT - volumetric modulated arc therapy; PTV - planning target volume; 6DoF - six-degrees of freedom

This is further illustrated in the DVH (see Figure 2) with the original VMAT plan (triangle) and simulated VMAT plan (square). The blue arrow shows the original intended coverage for all targets (> 95% of PTVs receiving 20 Gy dose). Loss of PTV (orange) coverages were observed up to 45% and loss of GTV (red) coverages of up to 25% in some lesions. OAR doses fluctuated depending on the random uncertainty induced to the simulated VMAT plans, and in some cases resulting in substantial increases to OAR doses. Figures 1 and 2 demonstrate the example case of an increased dose to the hippocampus (pink color). In the original plan, the maximal dose to hippocampi was kept below 6.3 Gy [13]. With induced random errors in the simulated VMAT plan, maximal dose to hippocampi was 8.1 Gy, exceeding the protocol requirement of 6.5 Gy. This was visible in the sagittal image of Figure 1 above, where the 50% isodose line (10 Gy) was perturbed towards the hippocampus (pink contour) and also shown in DVH (see figure 2).

**Figure 3:**
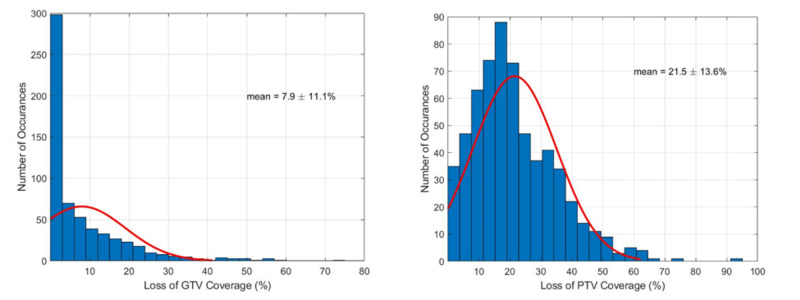
Probability distribution functions demonstrating the loss of coverage for both the GTVs (left) and PTVs (right) of all nine patients (61 tumors), randomly repeated each patient over 10 times (610 iterations) Residual set up errors between [± 2.0 mm, ± 2.0 deg] were applied in all 6DoF to replicate the day-to-day clinically representative residual set up errors. Due to these small setup errors, clinically unacceptable loss of target coverage was observed, which projects the magnitude of loss that would be seen in a typical single-isocenter VMAT treatment. VMAT - volumetric modulated arc therapy; 6DoF - six-degrees of freedom

An analysis of loss of coverage for all nine patients with setup uncertainty is displayed in Figure 3. The probability distribution function of coverage loss due to GTVs and the PTVs is shown. The number of occurrences of a particular coverage loss was binned in the histograms for all 10 iterations of the simulation for all nine patients (61 original targets, a total of 610 simulated targets). The red line represents the distributions correlating to the histogram with relative target coverage losses for GTVs and PTVs with the average values of 7.9 ± 11.1% and 21.5 ± 13.6%, respectively. This means for a typical single-isocenter VMAT plan, predictively, a loss of coverage of this magnitude would potentially exist during multiple brain metastases SRS treatment.

**Figure 4:**
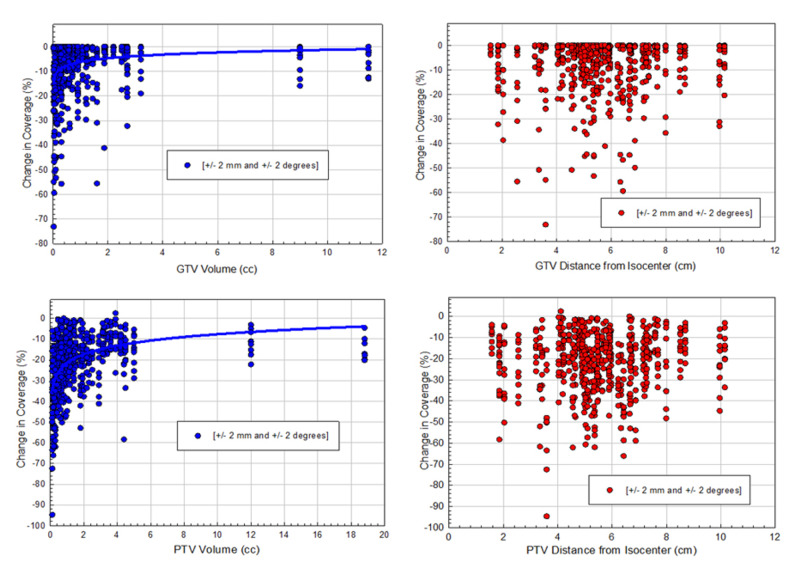
Scatter plots of the relative dose errors of target coverage due to random residual set up errors as a function of tumor volume and distance to isocenter for both GTVs (upper panel) and PTVs (lower panel) were shown Greater loss of target coverage for smaller tumor sizes was correlated for both GTVs and PTVs. However, no obvious trends were seen when comparing the loss of coverage and distance from the isocenter while simulating all 6DoF residual set up errors. GTV - gross tumor volume; PTV - planning target volume; 6DoF - six-degrees of freedom

The loss of target coverage due to random residual setup errors in 6DoF as a function of tumor size and distance to isocenter for both GTVs and PTVs are shown in Figure 4. A clear trend was evident that with a smaller GTV and PTV, there was an increased loss of target coverage (see left panel). However, there was no correlation between loss of coverage and distance to isocenter (right panel). This could be due to randomly generated clinically realistic translational error of ±2 mm (in each direction) dominating small but clinically observed rotational error of ±2° (in each direction). The variation in patient parameters such as tumor number, size, and distance to isocenter could make such a trend difficult to detect.

Regarding normal brain dose, no statistically significant difference was observed for MBD (p=0.41), V12 (p=0.97), V16 (p=0.79). However, in some cases, the maximal difference was up to 1.0 Gy, 5.2 cc, and 5.9 cc. In the LINAC-based SRS treatments, Blonigen et al. reported that normal brain V8 to V16 were the best predictors for radio-necrosis. Therefore, we caution that, in some cases, the maximal difference in V16 up to 5.9 cc could be detrimental to the normal brain [17]. Maximal dose increase of brainstem, optic apparatus and hippocampi were < 2.0 Gy, < 0.4 Gy, and < 2.7 Gy, respectively.

Simulated systematic errors

**Figure 5:**
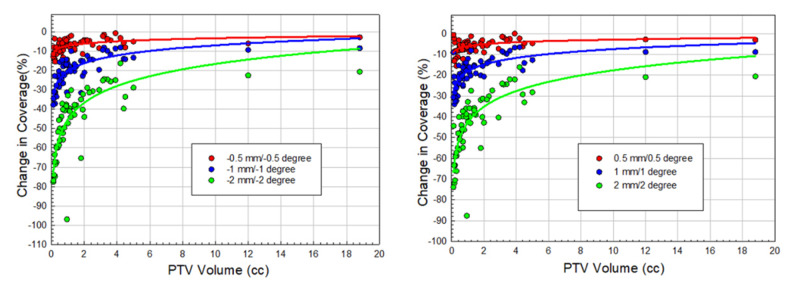
Scatter plots of loss of target coverage as a function of PTV volume is shown for all 61 targets All systematically induced errors were within [±2 mm, ±2 deg] in all 6DoF. There was a greater loss of target coverage for smaller tumors with the larger residual set up errors (green). PTV - planning target volume; 6DoF - six-degrees of freedom

To evaluate worst-case scenarios, we simulated single-isocenter VMAT plans with systematically assigned rotational and translational errors in all six-directions. This was performed by inducing systematic ±0.5 mm, ±1 mm, and ±2 mm, and ±0.5^o^, ±1^o^, and ±2^o^ errors in all 6DoF; results are shown in Figure 5. These simulations compared the results of the relative loss of PTV coverage for all 61 tumors. Loss of target coverage was a function of both the magnitude of induced uncertainty and tumor volume. The loss of target coverage due to systematic residual set up errors for 0.5 mm, 1 mm, and 2 mm, and 0.5^o^, 1^o^, and 2^o^ errors were 6.0 ± 3.1% (range, 0.5-15.6%), 18.2 ± 6.9% (range, 6.6-34.1%) and 42.9 ± 15.0% (range, 16.2-87.7%), respectively. Similar results were obtained for the negative direction (see Figure 5).

## Discussion

In this clinically representative simulation study, the dosimetric impact of clinically observable 6DoF residual setup errors in HyperArc style single-isocenter VMAT plans in the treatment of multiple brain metastases have been evaluated. Clinically unacceptable loss of target coverage for smaller tumors was observed when simulating residual isocenter misalignments. After applying clinically achievable random translational errors of ±2 mm and rotational errors of ±2° in each direction, the dramatic loss of the PTV coverage was observed with an average relative dose error of 21.5 ± 13.6% (up to 94.7% in some lesions) (p<0.001). This suggests that this technique could have major limitations in treating multiple brain lesions synchronously. The minimum dose to GTVs was lower by almost 4.0 Gy, on average, suggesting potential underdosing of small tumors.

Due to faster treatment delivery, LINAC-based VMAT SRS is becoming increasingly utilized, specifically using single-isocenter SRS [18]. The average beam-on time was less than six minutes. Varian developed a novel technology to automate this process using single-isocenter HyperArc VMAT for multiple lesions brain SRS [19]. Localization of each lesion is of the utmost importance when escalating dose to a small region. This is a difficult task, especially when treating multiple brain metastases simultaneously via a single-isocenter VMAT plan, including HyperArc VMAT. Patients are set up using a daily single CBCT to align bony anatomy; targets are not visible on daily CBCT, so targets are not localized individually. Small patient misalignments result in clinically significant loss of coverage and could deliver a very high dose adjacent to the tumor. This finding is of utmost importance because the HyperArc style single-isocenter VMAT could potentially deliver dosimetrically unacceptable treatments to patients through a near or complete geometric miss of the small brain lesions and increase damage to the adjacent critical structures. Major loss of dose conformity, lower GTV dose, higher relative dose errors for smaller lesions, and potentially higher doses to OAR’s including hippocampi and normal brain V12 and V16 have been demonstrated.

Other researchers have studied the loss of target coverage due to residual patient setup errors [9, 20-25]. Sagawa et al. reported that due to rotational setup errors, there was non-negligible underdosing of PTV coverage and a significant increase of normal brain V10 to V16 for multiple brain metastases patients with HA-VMAT plans [9]. Uncertainties were simulated using a third party software (MIM Maestro®, MIM Software Inc., USA) that could add additional source dosimetric errors [11]. Rotational setup errors were up to ± 3^o^ in each direction. Roper et al. systematically induced rotational errors of 0.5^o^, 1.0^o^, and 2.0^o^ using Velocity™ AI (Velocity Medical, Atlanta, USA) registration software [24]. They demonstrated a correlation between diminishing PTV coverage and distance from isocenter; however, their study was performed only using systematic rotational setup errors in single‐isocenter VMAT plans consisting of only two lesions. Their results showed that PTVD95% values worsened up to 60% of the prescribed dose in systematic rotations of 2° about all three axes. However, they did not include potential translational pretreatment set up errors and did not report the impact on OAR doses. In contrast, this study included all 6DoF residual set up errors for patients with multiple brain lesions (up to 16) and reported changes in OAR’s.

This investigative approach is more consistent with clinical realities by including all 6DoF residual patient setup errors and resulted in an even greater loss of target coverage than what other studies have shown. An innovative aspect of this study is that it uses computer-generated random uncertainties in all rotational and translational directions (in Eclipse) to apply to isocenter to simulate patient setup errors. This successfully simulates the types of errors encountered in a clinical setting without using third-party software, which avoids adding other sources of the dosimetric discrepancy. Another novelty in this study is the diversity of patients. Most similar studies used patients with few tumors, whereas patients in this study had up to 16 tumors (average of seven) and a total of 61 lesions. Unlike other studies, the simulations and treatment planning were all done within the Eclipse TPS, minimizing other error sources and making it more clinically representative. This tool can be used for both intracranial as well as extracranial multi-lesions and single-lesion stereotactic treatment settings.

There are some limitations of this study. First, due to the lack of a HyperArc planning license, HyperArc style single-isocenter VMAT plans were simulated. Although the arc geometry of HyperArc was kept the same, collimator optimizations were done manually to minimize the out of field dose on a per-patient basis. Second, this is a retrospective study of previously treated frame-based GK radiosurgery patients using high-resolution MRI imaging. Therefore, we did not have an appropriate planning CT imaging for heterogeneity corrections. For this simulation study, a homogenous dose to the brain was calculated, assigning a CT value equal to water. However, in a Monte Carlo study by Pokhrel et al. with fractionated-SRS treatments of cavernous sinus tumors, it was demonstrated that dose discrepancy is less than two percent in the brain with a pencil-beam algorithm [26]. Although a homogenous medium is used in routine GK radiosurgery treatments, we acknowledge that heterogeneity corrections will introduce most likely small dosimetric errors in the brain compared to these large discrepancies due to residual setup errors. In the future, planning CT images will be used for actual patient treatment via single-isocenter VMAT, co-registering MRI for tumors, and OAR delineation. In these results, a trend was not apparent with loss and distance to isocenter. Though geometrically, it should correlate, when calculating dose changes, other factors will affect this trend, such as dose gradient, depth, and SSD changes. Due to the proximity of multiple brain lesions and island blocking problems (two or more lesions sharing the same MLC pairs), the spread of 50% isodose lines created higher dose bridging in between/among the tumors. This created a major difficulty in calculating gradient indices for each lesion (results not shown here). Another limitation of the study is using standard MLCs of 5 mm width. Single-isocenter VMAT for multiple brain lesions is primarily limited to linear accelerators utilizing 2.5 mm high-definition MLCs. However, a recent study from Duke demonstrated that for radiosurgery of multiple brain metastases using a single-isocenter VMAT plan, 5 mm MLCs can produce similar target conformity with slightly increased 30-50% isodose spillage, but this can be minimized by adding one or two more VMAT arcs [27].

In summary, a single-isocenter VMAT treatment similar to HyperArc VMAT for multiple brain metastases can reduce treatment time significantly and improve treatment tolerability and clinic workflow. However, due to small but clinically relevant residual setup errors, an unacceptable loss of target coverage is observed. This could increase the dose to OAR, including the normal brain. Therefore, it is very important for any HyperArc style VMAT users to quantify these dosimetric discrepancies and develop correction strategies to minimize the dosimetric effects. Potential correction strategies are possible. First, institute an asymmetric margin around the GTV as a function of target volume and distance to isocenter ranging from one to three millimeters. Second, assign risk-adapted prescriptions up to 24 Gy (rather than 20 Gy) to small lesions away from the isocenter and no critical structures around the tumor. Third, create dual-isocenter VMAT plans, rather than single-isocenter plan, for a large number of multiple brain metastases as a function of distribution of the lesions in the brain, dividing the brain into two equal volumes. Any of these correction strategies could potentially compensate for the loss of target coverage and could be adopted on a patient-specific basis.

## Conclusions

Rapid treatment of multiple brain lesions using a single-isocenter VMAT is possible; however, small setup errors can result in large deviations from the planned target coverage, specifically for the smaller targets. This loss of target coverage due to small isocenter misalignment cannot be ignored for a single-isocenter VMAT plan, similar to the HyperArc VMAT plan. In some cases, large increases of normal brain dose V12 and V16 and maximal dose to OAR, including hippocampi, could be harmful. Further investigation of correction strategies is warranted.

## References

[1] Soffietti R, Ruda R, Mutani R (2002). Management of brain metastases. J Neurol.

[2] Eichler AF, Loeffler JS (2007). Multidisciplinary management of brain metastases. Oncologist.

[3] Chang EL (2008). Phase III Randomized clinical trial of radiosurgery with or without whole brain irradiation in patients newly diagnosed with 1 to 3 brain metastases. Int J Radiat Oncol Biol Phys.

[4] Hanna SA, Mancini A, Dal Col AH (2019). Frameless image-guided radiosurgery for multiple brain metastasis using VMAT: a review and an institutional experience. Front Oncol.

[5] Thomas EM, Popple RA, Wu X (2014). Comparison of plan quality and delivery time between volumetric arc therapy (RapidArc) and Gamma Knife radiosurgery for multiple cranial metastases. Neurosurgery.

[6] Kadoya N, Abe Y, Kajikawa T (2019). Automated noncoplanar treatment planning strategy in stereotactic radiosurgery of multiple cranial metastases: HyperArc and CyberKnife dose distributions. Med Dosim.

[7] Ruggieri R, Naccarato S, Mazzola R (2018). Linac-based VMAT radiosurgery for multiple brain lesions: comparison between a conventional multi-isocenter approach and a new dedicated mono-isocenter technique. Radiat Oncol.

[8] Ohira S, Ueda Y, Akino Y (2018). HyperArc VMAT planning for single and multiple brain metastases stereotactic radiosurgery: a new treatment planning approach. Radiat Oncol.

[9] Sagawa T, Ohira S, Ueda Y (2019). Dosimetric effect of rotational setup errors in stereotactic radiosurgery with HyperArc for single and multiple brain metastases. J Appl Clin Med Phys.

[10] Tryggestad E, Christian M, Ford E (2011). Inter- and intrafraction patient positioning uncertainties for intracranial radiotherapy: a study of four frameless, thermoplastic mask-based immobilization strategies using daily cone-beam CT. Int J Radiat Oncol Biol Phys.

[11] Clark GM, Fiveash JB, Prendergast BM (2011). Dosimetric impact of patient rotational setup errors with frameless single-isocenter, multi-target volumetric modulated arc radiosurgery for multiple brain metastases. Int J Radiat Oncol Biol Phys.

[12] Radiation Therapy Oncology Group (2011). A Phase II Trial of Hippocampal Avoidance During Whole Brain Radiotherapy for Brain Metastases, RTOG 0933. http://rpc.mdanderson.org/RPC/credentialing/files/RTOG-0933-Master[1].pdf.

[13] Birer SR, Olson AC, Adamson J (2017). Hippocampal dose from stereotactic radiosurgery for 4 to 10 brain metastases: risk factors, feasibility of dose reduction via re-optimization, and patient outcomes. Med Dosim.

[14] Marks LB, Ten Haken RK, Martel MK (2010). Quantitative analyses of normal tissue effects in the clinic. Int J Radiat Oncol Biol Phys.

[15] Minniti G, Clarke E, Lanzetta G (2011). Stereotactic radiosurgery for brain metastases: analysis of outcome and risk of brain radionecrosis. Radiat Oncol.

[16] Paddick I, Lippitz B (2006). A simple dose gradient measurement tool to complement the conformity index. J Neurosurg.

[17] Blonigen B, Steinmetz R D, Levin L (2010). Irradiated volume as a predictor of brain radionecrosis after linear accelerator stereotactic radiosurgery. Int J Radiat Oncol Biol Phys.

[18] Breneman J C, Steinmetz R, Smith A (2009). Frameless image-guided intracranial stereotactic radiosurgery: clinical outcomes for brain metastases. Int J Radiat Oncol Biol Phys.

[19] Alongi F, Fiorentino A, Gregucci F (2019). First experience and clinical results using a new non-coplanar mono-isocenter technique (HyperArc) for Linac-based VMAT radiosurgery in brain metastases. J Cancer Res Clin Oncol.

[20] Guckenberger M, Roesch J, Baier K (2012). Dosimetric consequences of translational and rotational errors in frame-less image-guided radiosurgery. Radiat Oncol.

[21] Chang J (2017). A statistical model for analyzing the rotational error of single isocenter for multiple targets technique. Med Phys.

[22] Stanhope C, Chang Z, Wang Z (2016). Physics considerations for single-isocenter, volumetric modulated arc radiosurgery for treatment of multiple intracranial targets. Pract Radiat Oncol.

[23] Kim S, Tseng TC, Morrow A (2015). Spatial variations of multiple off-axial targets for a single isocenter SRS treatment in Novalis Tx linac system. J Radiosurg SBRT.

[24] Roper J, Chanyavanich V, Betzel G, Switchenko J, Dhabaan A (2015). Single-isocenter multiple-target stereotactic radiosurgery: risk of compromised coverage. Int J Radiat Oncol Biol Phys.

[25] Stroom J, Vieira S, Mateus D (2014). On the robustness of VMAT-SABR treatment plans against isocentre positioning uncertainties. Radiat Oncol.

[26] Pokhrel D, Sood S, Badkul R, Jiang H, Saleh H, Wang F (2015). SU-E-T- 304: dosimetric comparison of cavernous sinus tumors: heterogeneity corrected pencil beam (PB-Hete) vs. X-ray Voxel Monte Carlo (XVMC) algorithms for stereotactic radiotherapy (SRT). Medical Physics.

[27] Abisheva Z, Floyd SR, Salama JK (2019). The effect of MLC leaf width in single-isocenter multi-target radiosurgery with volumetric modulated arc therapy. J Radiosurg SBRT.

